# Reference Values and Age and Sex Differences in Physical Performance Measures for Community-Dwelling Older Japanese: A Pooled Analysis of Six Cohort Studies

**DOI:** 10.1371/journal.pone.0099487

**Published:** 2014-06-12

**Authors:** Satoshi Seino, Shoji Shinkai, Yoshinori Fujiwara, Shuichi Obuchi, Hideyo Yoshida, Hirohiko Hirano, Hun Kyung Kim, Tatsuro Ishizaki, Ryutaro Takahashi, on behalf of the TMIG-LISA Research Group

**Affiliations:** 1 Tokyo Metropolitan Institute of Gerontology, 35-2 Sakae, Itabashi, Tokyo, Japan; 2 The Japan Society for the Promotion of Science, 8 Ichiban, Chiyoda, Tokyo, Japan; Marienhospital Herne - University of Bochum, Germany

## Abstract

**Objectives:**

To determine age- and sex-specific reference values for six physical performance measures, i.e. hand-grip strength, one-legged stance, and gait speed and step length at both usual and maximum paces, and to investigate age and sex differences in these measures among community-dwelling older Japanese adults.

**Methods:**

We conducted a pooled analysis of data from six cohort studies collected between 2002 and 2011 as part of the Tokyo Metropolitan Institute of Gerontology-Longitudinal Interdisciplinary Study on Aging. The pooled analysis included cross-sectional data from 4683 nondisabled, community-dwelling adults aged 65 years or older (2168 men, 2515 women; mean age: 74.0 years in men and 73.9 years in women).

**Results:**

Unweighted simple mean (standard deviation) hand-grip strength, one-legged stance, usual gait speed, usual gait step length, maximum gait speed, and maximum gait step length were 31.7 (6.7) kg, 39.3 (23.0) s, 1.29 (0.25) m/s, 67.7 (10.0) cm, 1.94 (0.38) m/s, and 82.3 (11.6) cm, respectively, in men and 20.4 (5.0) kg, 36.8 (23.4) s, 1.25 (0.27) m/s, 60.8 (10.0) cm, 1.73 (0.36) m/s, and 69.7 (10.8) cm, respectively, in women. All physical performance measures showed significant decreasing trends with advancing age in both sexes (all *P*<0.001 for trend). We also constructed age- and sex-specific appraisal standards according to quintiles. With increasing age, the sex difference in hand-grip strength decreased significantly (*P*<0.001 for age and sex interaction). In contrast, sex differences significantly increased in all other measures (all *P*<0.05 for interactions) except step length at maximum pace.

**Conclusion:**

Our pooled analysis yielded inclusive age- and sex-specific reference values and appraisal standards for major physical performance measures in nondisabled, community-dwelling, older Japanese adults. The characteristics of age-related decline in physical performance measures differed between sexes.

## Introduction

Physical performance measures (PPMs) such as usual gait speed and hand-grip strength are indicators not only of physical function, but also current and future overall well-being, in older adults [1], [2]. Recent systematic reviews and meta- and pooled analyses [3]–[6] showed that PPMs are effective at predicting adverse health outcomes, e.g. disability [7], institutionalization [8], hospitalization [9], and mortality [10]. A recent case-finding algorithm for sarcopenia [11] also included usual gait speed and hand-grip strength as appropriate screening tools. Thus, there is growing evidence of the importance of maintaining adequate physical performance in later life.

Some studies reported normative or reference values for PPMs [12]–[19]; however, no published study included age- and sex-specific reference values for multiple major PPMs among Asian adults or Japanese adults. Aoyagi et al. [20] conducted a cross-national comparison of PPMs in Japanese and American women and reported that gait speeds and chair stand times were faster for older Japanese women than for older American women, which suggests that traditional lifestyles may affect physical performance in later life. Because absolute levels of physical performance may vary between countries, it is difficult to extrapolate reference values from previous studies of Western populations [12]–[17], [19] to older Japanese people. Furthermore, the measuring protocols used for several PPMs, especially gait speed and hand-grip strength, varied considerably between studies and countries, which makes comparison of values difficult [21]–[24]. Therefore, age- and sex-specific PPM reference values specifically for older Japanese adults should be established using unified measuring protocols.

Collaborative research and the combining of cohort data have recently increased in the area of ageing studies [25]. Although the use of a cross-study approach allows analyses to encompass many geographic areas and much larger samples, there may be problems due to differences between studies in the measurement of variables and the protocols used. However, the Tokyo Metropolitan Institute of Gerontology-Longitudinal Interdisciplinary Study on Aging (TMIG-LISA) Research Group [7], [26]–[30] has regularly assessed one-legged stance with eyes open and usual and maximum gait step length, in addition to hand-grip strength and both usual and maximum gait speeds.

In the present study, we pooled cross-sectional data from cohort studies of the TMIG-LISA to establish reference values for six PPMs (hand-grip strength, one-legged stance with eyes open, and gait speed and step length at both usual and maximum paces), classified by age and sex. In addition, we investigated age and sex differences in these measures.

## Methods

### Data sources and study population

**Figure 1:**
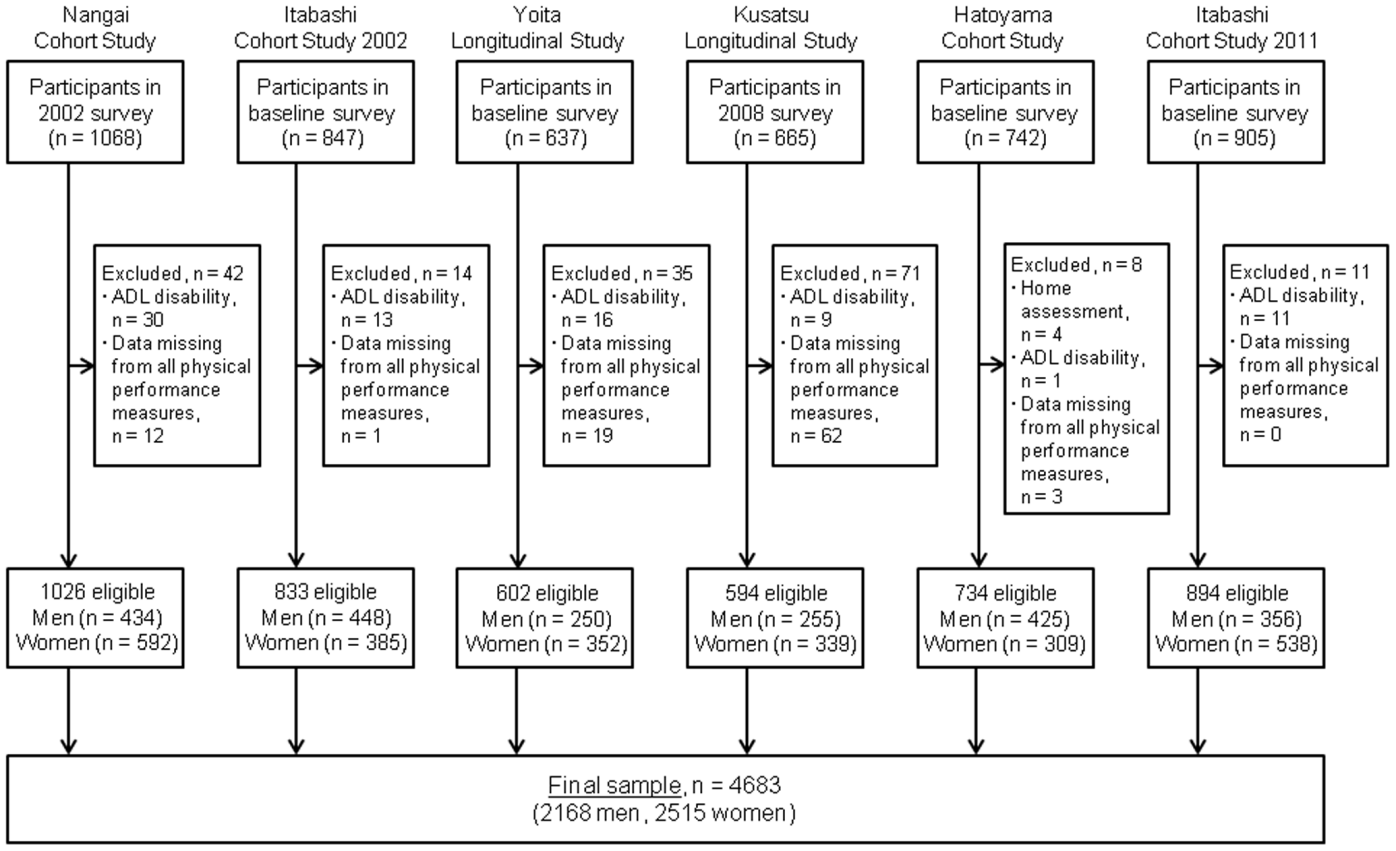
Schematic of participant selection processes in each included study.

The data sources for this study were derived from the TMIG-LISA [7], [26]–[30], which was established to determine risk factors for participants with geriatric diseases or chronic medical conditions and to identify factors that accelerate or decelerate aging in representative samples of older Japanese adults. In the present study, six TMIG cohort studies contributed data to a pooled analysis: the Nangai Cohort Study (NANGAI), Itabashi Cohort Study 2002 (ITABASHI02), Yoita Longitudinal Study (YOITA), Kusatsu Longitudinal Study (KUSATSU), Hatoyama Cohort Study (HATOYAMA), and Itabashi Cohort Study 2011 (ITABASHI11). We used baseline data or data from the year with the highest participation rate, all of which were collected between 2002 and 2011. The details of the study participants are discussed below (Figure 1).

#### Nangai Cohort Study (NANGAI)

Nangai village is a mainly agricultural area in the northern Japanese prefecture of Akita [31]. The baseline survey was held from July through August 1992, and the participant selection process is described in more detail elsewhere [7], [26], [28], [29]. In the present pooled analysis, we used surveillance data from 2002. The target population included 1327 residents (549 men, 778 women) aged 65 years or older. A total of 1068 ambulatory residents participated in the 2002 survey (446 men, participation rate of 81.2%; 622 women, participation rate of 79.9%; total participation rate of 80.5%).

#### Itabashi Cohort Study 2002 (ITABASHI02)

For ITABASHI02, a baseline survey was conducted in Itabashi ward in north-west Tokyo, Japan in 2002. Two thousand residents (1000 men, 1000 women) aged 71 years or older living in 36 residential areas in Itabashi ward were randomly recruited. After excluding 55 people who were institutionalized, 1945 invitations for the comprehensive health checkups were sent out. Ultimately, 847 residents participated in the baseline survey (456 men, participation rate of 45.6%; 391 women, participation rate of 39.1%; total participation rate of 43.5%).

#### Yoita Longitudinal Study (YOITA)

The Act on Assurance of Medical Care for Elderly People, which went into effect in Japan in 1983, requires all municipal governments in Japan to offer annual preventive health checkups to citizens aged 40 years or older. In conjunction with this service, we launched a longitudinal study on the aging and health of older adults living in Yoita town, a rural community in central Niigata Prefecture, Japan, in which older participants underwent an additional comprehensive geriatric assessment [32]. A total of 1380 residents (521 men, 859 women) aged 70 years or older were invited to participate in a baseline survey in 2004. Of those, 637 residents participated in the survey (261 men, participation rate of 50.1%; 376 women, participation rate of 43.8%; total participation rate of 46.2%).

#### Kusatsu Longitudinal Study (KUSATSU)

We also conducted a longitudinal study in Kusatsu town, a rural community in north-west Gunma Prefecture, Japan, in 2002 [32]. We used data from health checkups conducted in 2008. The study targeted National Health Insurance subscribers aged 65–74 years and individuals 75 years or older in the Medical Insurance System for the Elderly Aged 75 or Over (966 men, 1219 women). Of that population, 665 residents participated in the 2008 survey (276 men, participation rate of 28.6%; 389 women, participation rate of 31.9%; total participation rate of 30.4%).

#### Hatoyama Cohort Study (HATOYAMA)

The HATOYAMA study was a prospective cohort study of community-dwelling people aged 65 years or older living in the town of Hatoyama in Saitama Prefecture, Japan. The full details of the participant selection process were previously published [33]. Briefly, 2697 residents (1354 men, 1343 women) aged 65–84 years were selected using stratified sampling classified by age and residential area and random sampling strategies. Of those, 751 people participated in a baseline survey in 2010 (participation rate of 27.8%). Ultimately, 9 persons declined to participate in the study, and a total of 742 people were included in the study (428 men, participation rate of 31.6%; 314 women, participation rate of 23.4%; final participation rate of 27.5%).

#### Itabashi Cohort Study 2011 (ITABASHI11)

In the ITABASHI11 study, 7162 residents aged 65–84 years living in nine residential areas surrounding the TMIG were recruited in 2011. After excluding 463 people who were institutionalized or overlapped from previous studies, 6699 invitations (3136 men, 3563 women) for the health checkups were sent out. In October 2011, 913 ambulatory residents received health checkups (participation rate of 13.6%) [30]. Of those, 905 residents agreed to participate in the study (361 men, participation rate of 11.5%; 544 women, participation rate of 15.3%; final participation rate of 13.5%).

#### Final sample size

Of all the participants (n = 4864) in the pooled data of the present study, individuals were excluded if they were not independent in any of five basic activities of daily living (ADLs), i.e. bathing, dressing, walking, eating, and continence [7] or had data missing for all PPMs. We also excluded four participants in the HATOYAMA study because their PPMs were measured at their homes. The final, pooled sample size was 4683 (2168 men and 2515 women; 28.8% and 28.7%, respectively, of the target population). All participants provided written informed consent, and all studies included in the pooled analysis were conducted with the approval of the institutional review board and ethics committee of the TMIG.

### Assessment of health-related information

Age, body height and weight, history of chronic disease (hypertension, stroke, heart disease, and diabetes mellitus), self-rated health, alcohol drinking and smoking status, and Tokyo Metropolitan Institute of Gerontology Index of Competence (TMIG-IC) [34] were assessed in all cohorts. Body mass index (BMI) was defined as body weight divided by the height squared (kg/m^2^). History of chronic disease was determined through face-to-face interviews by physicians. Participants were asked whether a physician had diagnosed the specific condition (yes or no). Self-rated health (excellent, good, fair, or poor), alcohol drinking and smoking status (current, past, or never), and TMIG-IC were determined on the basis of questionnaire responses. The TMIG-IC was developed to assess levels of functional competence greater than those required for ADLs. The response to each item in this multidimensional 13-item index of competence is either ‘yes’ (able to perform) for 1 point or ‘no’ (unable to perform) for 0 points. The total score ranges from 0 to 13; lower scores indicate lower functional capacity [34].

### Assessment of PPMs

Well-trained staff measured hand-grip strength, one-legged stance with eyes open, and gait speed and step length at both usual and maximum paces. Participants wore the same type of shoe that had been prepared for them during the initial assessment.

#### Hand-grip strength

Hand-grip strength was assessed in all cohorts using common Smedley-type hand dynamometers [7], [29]. Participants stood with their arms hanging naturally at their sides holding the dynamometer with the grip size adjusted to a comfortable level. They were instructed and verbally encouraged to squeeze the hand-grip as hard as possible. In the YOITA, KUSATSU, and HATOYAMA studies, participants performed two trials with the dominant hand, and the best result (to the nearest 0.1 kg) was used. In all other cohorts, participants performed one trial with the dominant hand.

#### One-legged stance with eyes open

One-legged stance with eyes open was assessed using a participant's preferred leg in the NANGAI, KUSATSU, HATOYAMA, and ITABASHI11 studies. Participants were asked to place their hands at their waists while staring at a mark on the wall, raise one leg, and stand as long as possible. They were timed until they lost their balance or reached the maximum of 60 s [7]. Participants performed two trials, and the better time (to the nearest 0.1 s) was used.

#### Usual and maximum gait speeds

Usual and maximum gait speeds were measured over 5 m, with acceleration and deceleration phases of 3 m each, in all cohorts excepting ITABASHI11, in which participants were measured over a distance of 10 m, with acceleration and deceleration phases of 3 m each. Wang et al [35] reported that usual and maximum gait speeds measured over different distances are comparable only if acceleration and deceleration phases are used. We combined the 5 m and 10 m gait speeds because the acceleration and deceleration phases were identical for both measurement distances and because 3 m is considered sufficient to maintain steady usual and maximum gait speeds [35], [36].

Participants stood with their feet behind but just touching a starting line marked with tape at 0 m. Upon receiving the tester's command, they started walking at their normal and maximum paces along an 11-m (16-m in the ITABASHI11) course. The actual walking time was measured over 5 m, starting with the body trunk past the 3-m mark and ending with the body trunk after the 8-m (13-m in the ITABASHI11) mark [7], [29]. We calculated gait speed as distance divided by walking time (m/s). Usual gait speed was measured once. Maximum gait speed was measured twice, and the better of the two results (to the nearest 0.01 m/s) was used.

#### Usual and maximum gait step length

Step length is a component of gait speed and an independent predictor of cognitive decline [32]. Step length at both usual and maximum paces was assessed in the NANGAI, YOITA, KUSATSU, and HATOYAMA studies, in conjunction with usual and maximum gait speeds. Two other staff members measured mean step length by marking the heel points near the tape at 3 and 8 m and dividing the distance between the two heel points by the number of steps required [32]. Usual gait step length was measured once. Maximum gait step length was measured twice, and the better of the two results (to the nearest 0.1 cm) was used.

### Statistical analyses

We used descriptive statistics to characterize the study population. Differences in characteristics between men and women were analyzed using the unpaired *t* test, chi-square test, and Mann-Whitney *U* test. The means and standard deviations (SDs) of all PPMs were tabulated per 5-year age group (65–69, 70–74, 75–79, 80–84, and 85 years or older) for each sex. We also calculated gait speed and step length at both usual and maximum paces normalized for height (computed as speed or length divided by height in meters) because height is a predictor of gait speed [12]. Similarly, we normalized hand-grip strength for weight (computed as strength in kg divided by weight in kg). Furthermore, we performed a random effects meta-analysis using a Microsoft Excel spreadsheet developed by Neyeloff et al. [37] to obtain weighted means of PPMs and tested heterogeneity across studies using *Q* and *I* ^2^ statistics [38].

To evaluate linear trends in the means of PPMs between the age groups, we used weighted one-way analyses of variance by sex. Furthermore, we visualized univariate regression lines between age and PPMs in both sexes. To examine whether sex differences in PPMs changed with age (due to statistical interactions between age and sex), we performed multiple linear regression analyses with six PPMs as dependent variables, and age, sex (men  = 1, women  = 2), and the age × sex product terms as independent variables. In these analyses, we used the mean deviations of the independent variables to avoid issues related to multicolinearity [39]. In addition, one-legged stance with eyes open was log transformed.

Quintiles of each physical performance measure were used to construct appraisal standards according to sex and age group. We used an alpha level of 0.05 to identify statistical significance and performed all statistical analyses using IBM SPSS Statistics Version 20.

## Results

Table 1 shows the numbers of participants who provided complete data for each variable. Among the six PPMs, hand-grip strength and usual and maximum gait speed were assessed in all cohorts. The rates of missing data were 2.8% (n = 132) for hand-grip strength, 0.6% (n = 19) for one-legged stance with eyes open, 0.5% (n = 23) for usual gait speed, 0.7% (n = 22) for usual gait step length, 4.1% (n = 194) for maximum gait speed, and 3.9% (n = 111) for maximum gait step length. The lowest and highest rates of missing data were for usual and maximum gait speeds, respectively. The numbers of participants with complete data for each variable, by cohort, are available as (Table S1 in File S1).

**Table 1:** Numbers of participants with complete data for each variable.

Variables	Sample with complete data, n		
	Overall	Men	Women
	(n = 4683)	(n = 2168)	(n = 2515)
Age	4683	2168	2515
Height	4680	2165	2515
Weight	4681	2166	2515
Body mass index	4680	2165	2515
Chronic disease			
Hypertension	4674	2164	2510
Stroke	4674	2164	2510
Heart disease	4659	2158	2501
Diabetes mellitus	4677	2164	2513
Self-rated health	4681	2166	2515
Alcohol drinking status	4679	2165	2514
Smoking status	4677	2164	2513
TMIG-IC	4682	2167	2515
Physical performance measures			
Hand-grip strength	4551	2097	2454
One-legged stance with eyes open	3229	1463	1766
Usual gait speed	4660^a^	2154^c^	2506^e^
Usual gait step length	2934	1352	1582
Maximum gait speed	4489^b^	2075^d^	2414^f^
Maximum gait step length	2845	1326	1519

TMIG-IC =  Tokyo Metropolitan Institute of Gerontology Index of Competence.

a n = 3767 (5 m usual gait speed)+893 (10 m usual gait speed) = 4660.

b n = 3613 (5 m maximum gait speed)+876 (10 m maximum gait speed) = 4489.

c n = 1799 (5 m usual gait speed)+355 (10 m usual gait speed) = 2154.

d n = 1728 (5 m maximum gait speed)+347 (10 m maximum gait speed) = 2075.

e n = 1968 (5 m usual gait speed)+538 (10 m usual gait speed) = 2506.

f n = 1885 (5 m maximum gait speed)+529 (10 m maximum gait speed) = 2414.

Table 2 summarizes the characteristics of the study participants. There was no significant difference in age distribution between sexes. All PPM values were significantly higher in men than in women. The descriptive details of the study participants, by cohort (Tables S2 [men] and S3 [women] in File S1) and age group (Tables S4 [men] and S5 [women] in File S1), are available.

**Table 2:** Characteristics of the study participants (n = 4683).

Variables	Mean ± standard deviation or n (%)						*P* value
	Men (n = 2168)			Women (n = 2515)			
Age, years	74.0±5.3			73.9±5.5			0.800
Age group, n (%)							0.398
65–69	481(22.2)			588(23.4)			
70–74	727(33.5)			847(33.7)			
75–79	615(28.4)			658(26.2)			
80–84	297(13.7)			354(14.1)			
85 or over	48(2.2)			68(2.7)			
Geographic area, n (%)							<0.001
NANGAI	434(20.0)			592(23.5)			
ITABASHI02	448(20.7)			385(15.3)			
YOITA	250(11.5)			352(14.0)			
KUSATSU	255(11.8)			339(13.5)			
HATOYAMA	425(19.6)			309(12.3)			
ITABASHI11	356(16.4)			538(21.4)			
Height, cm	160.7±6.3			147.6±6.2			<0.001
Weight, kg	59.9±9.4			50.5±8.4			<0.001
Body mass index, kg/m^2^	23.2±3.0			23.2±3.5			0.917
Chronic disease, n (%)							
Hypertension	981(45.3)			1216(48.4)			0.033
Stroke	179(8.3)			104(4.1)			<0.001
Heart disease	459(21.3)			420(16.8)			<0.001
Diabetes mellitus	300(13.9)			232(9.2)			<0.001
Self-rated health, n (%)							<0.001
Excellent to good	1777(82.0)			1997(79.4)			
Fair to poor	389(18.0)			518(20.6)			
Alcohol drinking status, n (%)							<0.001
Current	1426(65.9)			581(23.1)			
Past	245(11.3)			137(5.4)			
Never	494(22.8)			1796(71.4)			
Smoking status, n (%)							<0.001
Current	526(24.3)			107(4.3)			
Past	1048(48.4)			130(5.2)			
Never	590(27.3)			2276(90.6)			
TMIG-IC, score (0–13)	12.0±1.6			12.0±1.7			0.527
Instrumental self-maintenance (0–5)	4.8±0.6			4.9±0.6			0.002
Intellectual activity (0–4)	3.7±0.7			3.5±0.9			<0.001
Social role (0–4)	3.5±0.8			3.7±0.7			<0.001
Physical performance measures							
Hand-grip strength, kg	31.7±6.7			20.4±5.0			<0.001
One-legged stance with eyes open, s	39.3±23.0			36.8±23.4			0.003
Usual gait speed, m/s	1.29±0.25			1.25±0.27			<0.001
Usual gait step length, cm	67.7±10.0			60.8±10.0			<0.001
Maximum gait speed, m/s	1.94±0.38			1.73±0.36			<0.001
Maximum gait step length, cm	82.3±11.6			69.7±10.8			<0.001

NANGAI =  Nangai Cohort Study; ITABASHI02 =  Itabashi Cohort Study 2002; YOITA =  Yoita Longitudinal Study; KUSATSU =  Kusatsu Longitudinal Study; HATOYAMA =  Hatoyama Cohort Study; ITABASHI11 =  Itabashi Cohort Study 2011; TMIG-IC =  Tokyo Metropolitan Institute of Gerontology Index of Competence.

Tables 3 and 4 present unweighted simple means and SDs for PPMs according to age group in men and women, respectively. In both sexes, the sample size was small for the age group 85 years or older, and all PPMs showed significant decreasing trends with advancing age (all *P*<0.001 for trend). Unweighted simple means for PPMs according to age group were very similar to and only slightly lower than weighted means (Table S6 in File S1). The *Q* statistics for all age strata had probability levels exceeding 0.05 (*I* ^2^ = 0.0–29.2%), indicating that studies were homogeneous within strata.

**Table 3:** Descriptive statistics for physical performance measures according to age group (men).

Variables	Mean ± standard deviation																		*P*
				Age group															for trend
	Overall			65–69			70–74			75–79			80–84			85 or over			
Hand-grip strength, kg	31.7	±	6.7	35.4	±	5.8	32.7	±	6.6	30.2	±	6.1	27.7	±	5.9	23.2	±	5.3	<0.001
Weight-adjusted hand-grip strength,	0.54	±	0.11	0.58	±	0.11	0.54	±	0.11	0.52	±	0.11	0.49	±	0.11	0.45	±	0.10	<0.001
[strength (kg)/weight (kg)]																			
(n)	(2097)			(473)			(699)			(597)			(281)			(47)			
One-legged stance with eyes open, s	39.3	±	23.0	46.9	±	20.6	41.7	±	21.9	33.2	±	22.9	26.0	±	22.5	21.9	±	23.5	<0.001
(n)	(1463)			(480)			(452)			(338)			(166)			(27)			
Usual gait speed, m/s	1.29	±	0.25	1.39	±	0.22	1.33	±	0.23	1.26	±	0.24	1.16	±	0.25	1.11	±	0.28	<0.001
Height-adjusted usual gait speed,	0.81	±	0.16	0.86	±	0.14	0.83	±	0.14	0.79	±	0.16	0.73	±	0.16	0.72	±	0.18	<0.001
[speed (m/s)/height (m)]																			
(n)	(2154)			(479)			(722)			(612)			(293)			(48)			
Usual gait step length, cm	67.7	±	10.0	71.4	±	8.1	69.6	±	9.2	65.4	±	9.8	60.7	±	9.9	57.3	±	11.3	<0.001
Height-adjusted usual gait step length,	42.3	±	5.9	44.1	±	5.0	43.3	±	5.4	41.3	±	6.2	38.6	±	6.1	37.2	±	6.8	<0.001
[length (cm)/height (m)]																			
(n)	(1352)			(389)			(444)			(322)			(149)			(48)			
Maximum gait speed, m/s	1.94	±	0.38	2.09	±	0.36	2.00	±	0.36	1.87	±	0.36	1.73	±	0.37	1.65	±	0.41	<0.001
Height-adjusted maximum gait speed,	1.21	±	0.23	1.29	±	0.22	1.24	±	0.22	1.17	±	0.22	1.09	±	0.23	1.07	±	0.26	<0.001
[speed (m/s)/height (m)]																			
(n)	(2075)			(468)			(697)			(588)			(275)			(47)			
Maximum gait step length, cm	82.3	±	11.6	86.8	±	9.2	84.6	±	10.6	79.2	±	11.1	73.9	±	12.0	70.7	±	13.2	<0.001
Height-adjusted maximum gait	51.4	±	6.8	53.6	±	5.5	52.7	±	6.2	49.9	±	6.8	46.9	±	7.4	45.8	±	8.0	<0.001
step length, [length (cm)/height (m)]																			
(n)	(1326)			(382)			(438)			(317)			(142)			(47)			

**Table 4:** Descriptive statistics for physical performance measures according to age group (women).

Variables	Mean ± standard deviation																		*P*
				Age group															for trend
	Overall			65–69			70–74			75–79			80–84			85 or over			
Hand-grip strength, kg	20.4	±	5.0	22.8	±	4.4	21.2	±	4.5	19.1	±	4.8	17.4	±	4.3	15.2	±	4.8	<0.001
Weight-adjusted hand-grip strength,	0.41	±	0.10	0.44	±	0.10	0.42	±	0.10	0.39	±	0.10	0.37	±	0.10	0.35	±	0.12	<0.001
[strength (kg)/weight (kg)]																			
(n)	(2454)			(581)			(822)			(645)			(345)			(61)			
One-legged stance with eyes open, s	36.8	±	23.4	48.5	±	18.7	38.9	±	22.7	28.4	±	22.4	20.0	±	19.9	10.6	±	15.2	<0.001
(n)	(1766)			(587)			(543)			(375)			(213)			(48)			
Usual gait speed, m/s	1.25	±	0.27	1.39	±	0.22	1.31	±	0.23	1.18	±	0.25	1.05	±	0.28	0.92	±	0.25	<0.001
Height-adjusted usual gait speed,	0.84	±	0.18	0.93	±	0.14	0.88	±	0.15	0.80	±	0.17	0.72	±	0.19	0.65	±	0.17	<0.001
[speed (m/s)/height (m)]																			
(n)	(2506)			(588)			(845)			(653)			(352)			(68)			
Usual gait step length, cm	60.8	±	10.0	65.4	±	7.4	63.3	±	8.4	58.1	±	9.9	54.1	±	10.1	48.1	±	9.9	<0.001
Height-adjusted usual gait step length,	41.5	±	6.2	43.9	±	4.7	42.9	±	5.2	40.1	±	6.5	37.7	±	6.7	34.2	±	6.4	<0.001
[length (cm)/height (m)]																			
(n)	(1582)			(448)			(490)			(374)			(202)			(68)			
Maximum gait speed, m/s	1.73	±	0.36	1.92	±	0.30	1.79	±	0.31	1.64	±	0.33	1.48	±	0.35	1.33	±	0.37	<0.001
Height-adjusted maximum gait speed,	1.17	±	0.23	1.28	±	0.19	1.20	±	0.20	1.12	±	0.22	1.02	±	0.23	0.94	±	0.25	<0.001
[speed (m/s)/height (m)]																			
(n)	(2414)			(577)			(817)			(629)			(332)			(59)			
Maximum gait step length, cm	69.7	±	10.8	74.8	±	8.1	71.5	±	9.1	67.3	±	10.5	62.0	±	11.3	56.3	±	11.6	<0.001
Height-adjusted maximum gait	47.5	±	6.8	50.1	±	5.1	48.5	±	5.7	46.3	±	7.0	43.1	±	7.5	39.9	±	7.6	<0.001
step length, [length (cm)/height (m)]																			
(n)	(1519)			(440)			(475)			(355)			(190)			(59)			

**Figure 2:**
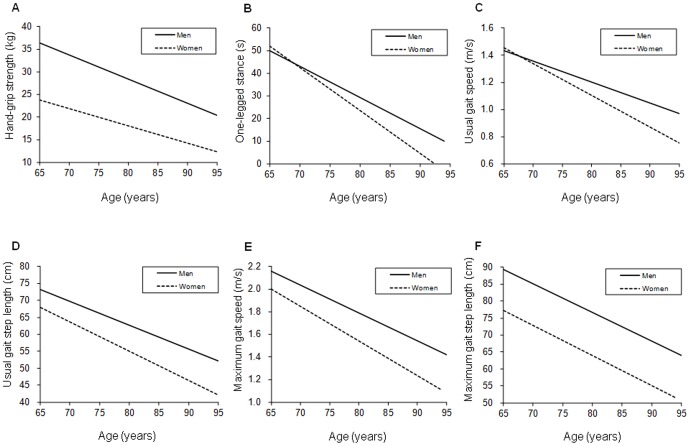
Univariate linear regression lines between age and physical performance (A–F) in men and women. Univariate linear regression analysis showed significant associations between age and all physical performance measures in both sexes (all *P*<0.001). **A** =  hand-grip strength, **B** =  one-legged balance with eyes open, **C** =  usual gait speed, **D** =  usual gait step length, **E** =  maximum gait speed, and **F** =  maximum gait step length.

Univariate linear regression analysis also showed significant associations between age and all PPMs in both sexes (all *P*<0.001; Figure 2). In multiple linear regression analyses, age and sex were significantly associated with hand-grip strength (standardized regression coefficient [*β*] = −0.30 and −0.70, respectively), one-legged stance with eyes open (*β* = −0.39 and −0.06, respectively), usual gait speed (*β* = −0.40 and −0.09, respectively) and step length (*β* = −0.42 and −0.31, respectively), and maximum gait speed (*β* = −0.39 and −0.27, respectively) and step length (*β* = −0.38 and −0.48, respectively) (all *P*<0.001). Age × sex interactions were small but significant in hand-grip strength (*β* = 0.05, *P*<0.001), one-legged stance with eyes open (*β* = −0.08, *P*<0.001), usual gait speed (*β* = −0.08, *P*<0.001) and step length (*β* = −0.03, *P* = 0.030), and maximum gait speed (*β* = −0.04, *P* = 0.002). However, the age × sex interaction was not significant for maximum gait step length (*β* = −0.01, *P* = 0.527). These associations and interactions remained significant after adjusting for chronic diseases, alcohol intake and smoking status.

Finally, Tables 5 and 6 show quintiles of PPMs according to age group in men and women, respectively. Although ceiling effects were seen in both sexes in the age group 65–74 years on the one-legged stance with eyes open test, all other PPMs had an approximately symmetrical distribution. The quintiles of weight-adjusted hand-grip strength and height-adjusted gait speed and step length at both usual and maximum paces are included as (Tables S7 [men] and S8 [women] in File S1).

**Table 5:** Quintiles of physical performance measures according to age group (men).

Physical performance measures	Quintile levels		Age					
			Overall	65–69	70–74	75–79	80–84	85 or over
Hand-grip strength, kg	5	(Highest)	38.0< =	40.0< =	39.0< =	35.0< =	32.0< =	27.0< =
	4		34.0–37.9	37.0–39.9	35.0–38.9	32.0–34.9	29.0–31.9	25.0–26.9
	3		30.0–33.9	34.0–36.9	31.0–34.9	29.0–31.9	26.0–28.9	22.0–24.9
	2		26.0–29.9	31.0–33.9	27.0–30.9	25.0–28.9	23.0–25.9	19.0–21.9
	1	(Lowest)	<26.0	<31.0	<27.0	<25.0	<23.0	<19.0
One-legged stance with eyes open, s	5	(Highest)	60.0< =	60.0< =	60.0< =	60.0< =	60.0< =	56.0< =
	4		60.0< =	60.0< =	60.0< =	42.0–59.9	28.0–59.9	15.0–55.9
	3		33.0–59.9	60.0< =	40.0–59.9	22.0–41.9	12.0–27.9	5.0–14.9
	2		10.0–32.9	25.0–59.9	15.0–39.9	7.0–21.9	4.0–11.9	3.0–4.9
	1	(Lowest)	<10.0	<25.0	<15.0	<7.0	<4.0	<3.0
Usual gait speed, m/s	5	(Highest)	1.49< =	1.56< =	1.52< =	1.47< =	1.36< =	1.31< =
	4		1.35–1.48	1.43–1.55	1.39–1.51	1.32–1.46	1.25–1.35	1.15–1.30
	3		1.25–1.34	1.35–1.42	1.28–1.38	1.20–1.31	1.11–1.24	1.08–1.14
	2		1.11–1.24	1.22–1.34	1.16–1.27	1.10–1.19	0.96–1.10	0.90–1.07
	1	(Lowest)	<1.11	<1.22	<1.16	<1.10	<0.96	<0.90
Usual gait step length, cm	5	(Highest)	76.0< =	78.0< =	76.0< =	73.0< =	68.0< =	66.0< =
	4		71.0–75.9	73.0–77.9	71.0–75.9	69.0–72.9	64.0–67.9	61.0–65.9
	3		66.0–70.9	70.0–72.9	68.0–70.9	64.0–68.9	59.0–63.9	54.0–60.9
	2		60.0–65.9	65.0–69.9	63.0–67.9	58.0–63.9	52.0–58.9	48.0–53.9
	1	(Lowest)	<60.0	<65.0	<63.0	<58.0	<52.0	<48.0
Maximum gait speed, m/s	5	(Highest)	2.27< =	2.38< =	2.27< =	2.17< =	2.00< =	1.91< =
	4		2.00–2.26	2.17–2.37	2.08–2.26	1.92–2.16	1.85–1.99	1.80–1.90
	3		1.85–1.99	2.00–2.16	1.92–2.07	1.80–1.91	1.67–1.84	1.61–1.79
	2		1.68–1.84	1.86–1.99	1.72–1.91	1.61–1.79	1.45–1.66	1.32–1.60
	1	(Lowest)	<1.68	<1.86	<1.72	<1.61	<1.45	<1.32
Maximum gait step length, cm	5	(Highest)	91.0< =	93.0< =	93.0< =	88.0< =	83.0< =	81.0< =
	4		85.0–90.9	88.0–92.9	87.0–92.9	83.0–87.9	78.0–82.9	73.0–80.9
	3		81.0–84.9	85.0–87.9	82.0–86.9	78.0–82.9	72.0–77.9	68.0–72.9
	2		75.0–80.9	80.0–84.9	78.0–81.9	71.0–77.9	63.0–71.9	62.0–67.9
	1	(Lowest)	<75.0	<80.0	<78.0	<71.0	<63.0	<62.0

**Table 6:** Quintiles of physical performance measures according to age group (women).

Physical performance measures	Quintile levels		Age					
			Overall	65–69	70–74	75–79	80–84	85 or over
Hand-grip strength, kg	5	(Highest)	24.0< =	26.0< =	25.0< =	23.0< =	21.0< =	20.0< =
	4		22.0–23.9	24.0–25.9	23.0–24.9	20.0–22.9	19.0–20.9	16.0–19.9
	3		19.0–21.9	22.0–23.9	20.0–22.9	18.0–19.9	17.0–18.9	14.0–15.9
	2		16.0–18.9	19.1–21.9	18.0–19.9	15.0–17.9	14.0–16.9	11.0–13.9
	1	(Lowest)	<16.0	<19.0	<18.0	<15.0	<14.0	<11.0
One-legged stance with eyes open, s	5	(Highest)	60.0< =	60.0< =	60.0< =	60.0< =	39.0< =	17.0< =
	4		60.0< =	60.0< =	60.0< =	30.0–59.9	17.0–38.9	5.0–16.9
	3		26.0–59.9	60.0< =	31.0–59.9	14.0–29.9	8.0–16.9	3.0–4.9
	2		9.0–25.9	29.0–59.9	12.0–30.9	6.0–13.9	3.0–7.9	2.0–2.9
	1	(Lowest)	<9.0	<29.0	<12.0	<6.0	<3.0	<2.0
Usual gait speed, m/s	5	(Highest)	1.47< =	1.55< =	1.52< =	1.39< =	1.28< =	1.15< =
	4		1.32–1.46	1.43–1.54	1.39–1.51	1.25–1.38	1.14–1.27	0.94–1.14
	3		1.20–1.31	1.34–1.42	1.27–1.38	1.14–1.24	1.00–1.13	0.83–0.93
	2		1.05–1.19	1.22–1.33	1.14–1.26	0.98–1.13	0.80–0.99	0.70–0.82
	1	(Lowest)	<1.05	<1.22	<1.14	<0.98	<0.80	<0.70
Usual gait step length, cm	5	(Highest)	69.0< =	71.0< =	70.0< =	66.0< =	63.0< =	56.0< =
	4		64.0–68.9	67.0–70.9	66.0–69.9	61.0–65.9	57.0–62.9	50.0–55.9
	3		60.0–63.9	64.0–66.9	62.0–65.9	57.0–60.9	51.0–56.9	45.0–49.9
	2		53.0–59.9	60.0–63.9	57.0–61.9	51.0–56.9	46.0–50.9	40.0–44.9
	1	(Lowest)	<53.0	<60.0	<57.0	<51.0	<46.0	<40.0
Maximum gait speed, m/s	5	(Highest)	2.00< =	2.13< =	2.04< =	1.89< =	1.79< =	1.66< =
	4		1.85–1.99	2.00–2.12	1.85–2.03	1.72–1.88	1.61–1.78	1.40–1.65
	3		1.67–1.84	1.85–1.99	1.72–1.84	1.59–1.71	1.39–1.60	1.20–1.39
	2		1.47–1.66	1.72–1.84	1.56–1.71	1.39–1.58	1.21–1.38	0.96–1.19
	1	(Lowest)	<1.47	<1.72	<1.56	<1.39	<1.21	<0.96
Maximum gait step length, cm	5	(Highest)	78.0< =	81.0< =	79.0< =	75.0< =	72.0< =	67.0< =
	4		73.0–77.9	76.0–80.9	74.0–78.9	70.0–74.9	65.0–71.9	59.0–66.9
	3		69.0–72.9	73.0–75.9	70.0–73.9	65.0–69.9	59.0–64.9	54.0–58.9
	2		62.0–68.9	70.0–72.9	65.0–69.9	60.0–64.9	53.0–58.9	47.0–53.9
	1	(Lowest)	<62.0	<70.0	<65.0	<60.0	<53.0	<47.0

## Discussion

### Main findings

Our pooled analysis established age- and sex-specific unweighted simple mean values for six PPMs among nondisabled, community-dwelling, older Japanese adults. Our study populations from six cohort studies were homogeneous. In addition, unweighted simple means for PPMs from a pooled analysis were very similar to weighted means from a random effects meta-analysis model, and their 95% confidence intervals largely overlapped. Therefore, we used unweighted simple means as the reference values and also constructed age- and sex-specific appraisal standards according to quintiles. These reference values and appraisal standards can be used in comparative assessments of healthy Japanese of the same sex and age group.

Sex difference in hand-grip strength significantly decreased with increasing age. In contrast, sex differences significantly increased for one-legged stance with eyes open, usual gait speed and step length, and maximum gait speed. These results suggest there are sex differences in the age-related decline of PPMs.

### Comments on our results

#### Reference values

The present study is the first to report age- and sex-specific values for both gait speed and step length at usual and maximum paces in older Japanese adults. Usual gait speed and hand-grip strength are the most commonly examined measures worldwide [5], and individual normative and reference data have been published, most commonly usual gait speed [12], [16], [17], [19], [40], [41]. However, several studies [7], [32], [42], [43] reported that maximum gait speed and step length at both usual and maximum paces were also valid for predicting adverse health outcomes. Shinkai et al. [7] reported that usual gait speed was more sensitive in predicting onset of ADL disability among people aged 75 years or older, whereas maximum gait speed was more sensitive among people aged 65–74 years. Fitzpatrick et al. [42] reported that maximum gait speed was most sensitive in predicting early cognitive decline in a healthy cohort. Furthermore, Taniguchi et al. [32] showed that usual gait step length in women and maximum gait step length in men were better than either usual or maximum gait speed at predicting future cognitive decline. These results indicate that measuring gait performance at both usual and maximum paces is important because the ability to voluntarily increase gait performance, i.e. gait speed and step length, may better reflect individual reserves in overall health status. Moreover, measuring gait parameters such as step length, cadence, and variability during maximum walking may optimize detection of early cognitive dysfunction among healthy older people [32], [44]. Unfortunately, reference values for these measures were not previously available. This study is of great significance as it provides inclusive reference values for the PPMs considered to be the best indicators of overall well-being.

Compared with previous study results from Western populations [6], [40], [41], [45], the mean values in the present study tend to be somewhat lower for hand-grip strength and higher in gait speed and step length. The measurement protocol for hand-grip strength recommended by The American Society of Hand Therapists (ASHT) [46] has been widely used in Western countries. That protocol calls for participants to be seated, shoulders adducted and neutrally rotated, elbow flexed at 90°, forearm in a neutral position, and the wrist between 0 and 30° of dorsiflexion. However, the protocol of standing with fully extended elbows has been used throughout Japan [7], and the standing protocol produced higher values than the ASHT recommended position [21]. Nevertheless, hand-grip strength was higher in older Westerners [45] than in the older Japanese included in the present study, which suggests that differences in body type (older Japanese are thinner and have less muscle mass than Westerners [47]) have a stronger effect than differences in measuring protocols.

Regarding the difference in gait performance between Western and Japanese adults, a component of the traditional Japanese lifestyle, i.e. lifelong squatting behaviors, may have a long-term effect on the ability to sit, squat, and rise from floor level to a standing position [20]. However, a more important factor in the discrepancy in gait performance may be the difference in measuring protocols used in the Western and Japanese studies. In Western studies, a “static-start” protocol, whereby the individual stands at the starting line, and timing begins with a verbal “go” command, is more common [23]. However, in Japan, gait speed is measured over a 5 m, with acceleration and deceleration phases of 3 m each [7], i.e. a “dynamic-start” protocol [23]. The gait speed at usual and maximum paces measured by dynamic-start protocols was significantly faster than that measured by static-start protocols [35]. These differences should be considered when values for PPMs are compared between Western and Japanese adults.

#### Age and sex differences

Interestingly, although the absolute levels of PPMs in the present study were somewhat different from those in Western populations, the identified age and sex differences were consistent with those in previous studies [25], [48]. A decrease in the sex difference in hand-grip strength with increasing age is fairly common [25], [48], [49]. Moreover, most studies reported higher gait performance in men than in women [12], [15]–[19], comparable to our results. When usual gait speed and step length were normalized to height, the sex difference in usual gait speed was inverted (normalized usual gait speed in women exceeded that in men), whereas usual gait step length was still longer in men. This suggests that men tend to walk with longer strides but lower cadences and women tend to walk with higher cadences, especially in younger age groups. These results have interesting implications and are consistent with the findings of a previous study [50]. Thus, populations may be similar in how physical performance changes with aging in men and women. Such sex differences in physical performance levels are likely partially due to differences in body size and/or body composition [51]; thus, future research in this area is needed.

The Asian Working Group on Sarcopenia [11] developed a case-finding algorithm for sarcopenia that recommends measuring muscle mass in older adults with slow gait speeds (< = 0.8 m/s) and/or with low hand-grip strength (<26 kg in men, <18 kg in women). Both measures were also included as surrogate markers of a frailty phenotype [52]. These operational definitions are used as across-the-board criteria regardless of age. However, age-related decline in PPMs is inevitable, even in a nondisabled older population, as shown in the present study. In addition, it was obvious that there were age and sex differences in physical performance and that absolute levels of PPMs vary between countries. Therefore, age- and sex-based PPM criteria specific to sarcopenia or frailty in Japanese should be defined in the future. For example, Verghese et al. [53] defined motoric cognitive risk syndrome as a value 1 SD below age- and sex-specific mean usual gait speeds.

### Uniformity of measuring protocols

The uniformity of measuring protocols within our pooled analysis may be better than in previous meta-analyses because there were few differences in measuring techniques across the studies. Such differences can limit comparability. Although the number of assessments of hand-grip strength differed according to cohort (one or two trials) in the present study, a previous study reported similar test-retest reliability after only one trial, the mean of two or three trials, and a maximum of three trials [54]. Coldham et al. [55] also found that one trial was as reliable and less tiring than three trials.

We analyzed gait speed by combining 5-m and 10-m gait speed measurements because different distances are comparable if acceleration and deceleration phases are used [35]. The means (±SD) of usual (1.29±0.25 m/s in men, 1.25±0.27 m/s in women) and maximum (1.94±0.38 m/s in men, 1.73±0.36 m/s in women) 5- and 10-m gait speeds combined were not substantially different from those of usual (1.29±0.25 m/s in men, 1.22±0.28 m/s in women) and maximum (1.94±0.38 m/s in men, 1.71±0.37 m/s in women) 5-m gait speeds (data not shown). However, we recommend a distance of 5 m with acceleration and deceleration phases of 3 m (> = 2.5 m [36]) each to assess steady-state gait speed, since more space is needed for a measuring distance of 10 m.

### Strengths and limitations

A strength of this study is the large sample size achieved by combining data from six cohorts. Generally, there tends to be fewer male participants than female participants in population-based studies; however, our rates of participation of the target population was similar between men (28.8%) and women (28.7%). Moreover, having a large number of randomly recruited male participants, such as in the ITABASHI02 and HATOYAMA studies, strengthens our analyses for age- and sex-specific reference values. At the present stage, there is no better representative data that is applicable for older Japanese adults. Our results can be used as the best guess in terms of reference values.

In contrast, the main limitation in this study was selection bias. The total participation rate in health checkups in our study was approximately 30% of the target population. Significant factors associated with non-participation in these community-based health checkups in older Japanese adults were low mental and physical functions such as cognitive dysfunction, low self-rated health and instrumental ADL, and mobility limitation [56]–[58]. Thus, relatively healthier people tend to participate. More specifically, the age group 85 years or older encompassed a relatively small sample size in both sexes. There may be a healthy volunteer effect in the strata. Practically speaking, since older people who are similar to our study population are the ones who participate in community-based health checkups and interventions, our reference values will be applicable to them. However, our findings might not be generalizable to older adults who are more frail. Finally, causality cannot be inferred regarding age and sex differences in PPMs due to the cross-sectional design of the study.

## Conclusions

This pooled analysis yielded age- and sex-specific reference values and appraisal standards for six PPMs in nondisabled, community-dwelling, older Japanese adults. Although absolute physical performance levels vary among populations, the characteristics of age and sex differences in PPMs may be broadly shared.

## Supporting Information

File S1 Contains Table S1, Numbers of participants with complete data, by variable and cohort. NANGAI =  Nangai Cohort Study; ITABASHI02 =  Itabashi Cohort Study 2002; YOITA =  Yoita Longitudinal Study; KUSATSU =  Kusatsu Longitudinal Study; HATOYAMA =  Hatoyama Cohort Study; ITABASHI11 =  Itabashi Cohort Study 2011; TMIG-IC =  Tokyo Metropolitan Institute of Gerontology Index of Competence. Table S2, Characteristics of male participants according to cohort. ^a^ gait speed measured over 5 m, ^b^ gait speed measured over 10 m, *one-way analysis of variance or χ^2^ test. NANGAI =  Nangai Cohort Study; ITABASHI02 =  Itabashi Cohort Study 2002; YOITA =  Yoita Longitudinal Study; KUSATSU =  Kusatsu Longitudinal Study; HATOYAMA =  Hatoyama Cohort Study; ITABASHI11 =  Itabashi Cohort Study 2011; TMIG-IC =  Tokyo Metropolitan Institute of Gerontology Index of Competence. Table S3, Characteristics of female participants according to cohort. ^a^ gait speed measured over 5 m, ^b^ gait speed measured over 10 m, *one-way analysis of variance or χ^2^ test. NANGAI =  Nangai Cohort Study; ITABASHI02 =  Itabashi Cohort Study 2002; YOITA =  Yoita Longitudinal Study; KUSATSU =  Kusatsu Longitudinal Study; HATOYAMA =  Hatoyama Cohort Study; ITABASHI11 =  Itabashi Cohort Study 2011; TMIG-IC =  Tokyo Metropolitan Institute of Gerontology Index of Competence. Table S4, Characteristics of male participants according to age group. *weighted one-way analysis of variance or χ^2^ test. NANGAI =  Nangai Cohort Study; ITABASHI02 =  Itabashi Cohort Study 2002; YOITA =  Yoita Longitudinal Study; KUSATSU =  Kusatsu Longitudinal Study; HATOYAMA =  Hatoyama Cohort Study; ITABASHI11 =  Itabashi Cohort Study 2011; TMIG-IC =  Tokyo Metropolitan Institute of Gerontology Index of Competence. Table S5, Characteristics of female participants according to age group. *weighted one-way analysis of variance or χ^2^ test. NANGAI =  Nangai Cohort Study; ITABASHI02 =  Itabashi Cohort Study 2002; YOITA =  Yoita Longitudinal Study; KUSATSU =  Kusatsu Longitudinal Study; HATOYAMA =  Hatoyama Cohort Study; ITABASHI11 =  Itabashi Cohort Study 2011; TMIG-IC =  Tokyo Metropolitan Institute of Gerontology Index of Competence. Table S6, Weighted means of physical performance measures obtained from a random effects meta-analysis model according to sex and age group across all studies. CI =  confidence interval. All *P* values for Cochran's *Q* statistic exceed 0.05. Table S7. Quintiles of weight-adjusted hand-grip strength and height-adjusted gait speed and step length according to age group (men). Table S8, Quintiles of weight-adjusted hand-grip strength and height-adjusted gait speed and step length according to age group (women).
(XLSX)
